# Polygenic Risk and the Course of Attention-Deficit/Hyperactivity Disorder From Childhood to Young Adulthood: Findings From a Nationally Representative Cohort

**DOI:** 10.1016/j.jaac.2020.12.033

**Published:** 2021-09

**Authors:** Jessica C. Agnew-Blais, Daniel W. Belsky, Avshalom Caspi, Andrea Danese, Terrie E. Moffitt, Guilherme V. Polanczyk, Karen Sugden, Jasmin Wertz, Benjamin S. Williams, Cathryn M. Lewis, Louise Arseneault

**Affiliations:** a King’s College London, United Kingdom; b South London and Maudsley NHS Foundation Trust, London, United Kingdom; c Columbia Mailman School of Public Health, New York, New York; d Duke University, Durham, North Carolina; e University of São Paulo Medical School, Brazil

**Keywords:** ADHD, development, longitudinal, polygenic risk score

## Abstract

**Objective:**

To understand whether genetic risk for attention-deficit/hyperactivity disorder (ADHD) is associated with the course of the disorder across childhood and into young adulthood.

**Method:**

Participants were from the Environmental Risk (E-Risk) Longitudinal Twin Study, a population-based birth cohort of 2,232 twins. ADHD was assessed at ages 5, 7, 10, and 12 with mother- and teacher-reports and at age 18 with self-report. Polygenic risk scores (PRSs) were created using a genome-wide association study of ADHD case status. Associations with PRS were examined at multiple points in childhood and longitudinally from early childhood to adolescence. We investigated ADHD PRS and course to young adulthood, as reflected by ADHD remission, persistence, and late onset.

**Results:**

Participants with higher ADHD PRSs had increased risk for meeting ADHD diagnostic criteria (odds ratios ranging from 1.17 at age 10 to 1.54 at age 12) and for elevated symptoms at ages 5, 7, 10, and 12. Higher PRS was longitudinally associated with more hyperactivity/impulsivity (incidence rate ratio = 1.18) and inattention (incidence rate ratio = 1.14) from age 5 to age 12. In young adulthood, participants with persistent ADHD exhibited the highest PRS (mean PRS = 0.37), followed by participants with remission (mean PRS = 0.21); both groups had higher PRS than controls (mean PRS = −0.03), but did not significantly differ from one another. Participants with late-onset ADHD did not show elevated PRS for ADHD, depression, alcohol dependence, or marijuana use disorder.

**Conclusion:**

Genetic risk scores derived from case-control genome-wide association studies may have relevance not only for incidence of mental health disorders, but also for understanding the longitudinal course of mental health disorders.

Genome-wide association studies (GWASs) of attention-deficit/hyperactivity disorder (ADHD) have begun to uncover the molecular basis of genetic risk for the disorder.^1^ A key question is whether genetic liability captured by GWASs is associated not only with case-control status, but also with different courses of ADHD across development. ADHD is not a static childhood disorder but can take different courses across the life span: some people will experience remission in childhood or adolescence, while others will continue to experience symptoms and impairment as adults. Meta-analysis finds about 15% of people with childhood ADHD continue to meet full diagnostic criteria into their mid-20s, with an additional 50% experiencing functional impairment.^2^ Recent research also suggests that some people experience late-onset ADHD, in which diagnostic criteria are met only after childhood.^3^, ^4^, ^5^ People whose ADHD persists or presents in adulthood have a range of adverse outcomes, including lower educational attainment, increased mental health disorders, and worse physical health.^6^^,^^7^ Despite the poor prognosis associated with these developmental courses of ADHD, relatively few predictors of change in ADHD across development have been identified.^8^ The aim of this study was to investigate whether genetic risk for ADHD, as captured by polygenic risk score (PRS), is associated with differing ADHD course across childhood and into young adulthood.

Studies that leverage shared genetics between family members point to heritable factors playing a role in ADHD developmental course.^9^ For example, twin studies report novel genetic influences on hyperactivity/impulsivity symptoms in adolescence independent of those in early childhood.^10^ A key question is whether findings from recent advances in molecular genetic studies can lead to a better understanding of how genetic risk affects ADHD developmental course. PRSs derived from GWASs are one way to capture genetic liability to ADHD. These scores have been shown to explain about 5% of the variance of ADHD case-control status in independent samples^1^ and are associated with dimensional measures of ADHD symptoms in both clinical and population-based studies.^11^, ^12^, ^13^ However, less is known about how ADHD PRS is associated with the longitudinal course of the disorder and whether people with a high genetic burden, as reflected in higher PRSs, may be more likely to show a more persistent course.

Recent research in population-based cohorts has raised the possibility of a novel ADHD course: late-onset ADHD. Late-onset ADHD occurs when people meet ADHD criteria only after the *DSM-5* age cutoff of 12 years and is controversial given the definition of ADHD as a childhood-onset neurodevelopmental disorder.^4^, ^5^, ^6^ Several explanations for late-onset ADHD have been proposed. Late onset could occur if ADHD was missed or subthreshold in childhood; alternatively, late-onset ADHD may be accounted for by symptoms of other disorders being misdiagnosed as ADHD.^14^^,^^15^ Genetics can help to disentangle these potential explanations. If late-onset ADHD is similar to childhood ADHD except for going unnoticed in early life, ADHD PRSs among the late-onset group would be similar to PRSs of people with apparent childhood ADHD. Conversely, if the late-onset group is accounted for by symptoms of other mental health disorders misattributed to ADHD, the late-onset group may show higher genetic risk for these other mental health disorders.^3^ Investigating genetic risk for mental health disorders that are commonly comorbid with ADHD can also help in understanding ADHD persistence. One of the few identified predictors of ADHD persistence is comorbidity with other mental health disorders^8^; thus, genetic risk for other mental health disorders may worsen ADHD prognosis among people with childhood ADHD.

In the current study, we investigated associations between ADHD genetic risk, as captured by the PRS, and ADHD course in a population-based longitudinal cohort. First, we examined how genetic risk was associated with ADHD diagnosis and symptoms in childhood at multiple ages ranging from early childhood (age 5) to early adolescence (age 12), from the perspective of different informants (mothers and teachers), and across symptom domains (hyperactivity/impulsivity and inattention). Second, we examined the longitudinal course of hyperactivity/impulsivity and inattention across childhood to assess whether ADHD PRS was associated with level of, and change in, ADHD symptoms across development. Third, we extended analyses to young adulthood to examine whether ADHD PRS was associated with persistence, remission, and late onset of ADHD from childhood to age 18. Fourth, we investigated whether genetic risk for other mental health disorders commonly comorbid with ADHD (depression, alcohol dependence, and marijuana use disorder) were associated with ADHD course, either through contributing to persistence or influencing risk for late-onset ADHD.

## Method

### Study Cohort

Participants were members of the Environmental Risk (E-Risk) Longitudinal Twin Study, a birth cohort of 2,232 British children drawn from a larger birth register of twins born in England and Wales in 1994–1995.^16^ Full details about the sample are reported in Supplement 1, available online.^17^ Briefly, the E-Risk sample was constructed in 1999–2000 when 1,116 families with same-sex 5-year-old twins participated in home-visit assessments. This sample comprised 56% monozygotic and 44% dizygotic twin pairs; sex was evenly distributed within zygosity (49% male). Families were recruited to represent the population of the United Kingdom with newborns in the 1990s on the basis of residential location throughout England and Wales and mother’s age. At follow-up, the sample represented the full range and prevalence rates of socioeconomic levels in the United Kingdom (see Figure S1, available online).^18^ Follow-up home visits were conducted when children were age 7 years (98% participation), age 10 years (96% participation), age 12 years (96% participation), and age 18 years (93% participation). Home visits at ages 5–12 years included assessments with participants and their mother; we conducted full interviews with participants only at age 18 (n = 2,066). There were no differences between participants who did and did not take part at age 18 in socioeconomic status (SES) when the cohort was initially defined (χ^2^ = 0.86, d.f. = 2, *p* = .65), age 5 IQ (*t* = 0.98, d.f. = 2208, *p* = .33), or rates of childhood ADHD (χ^2^ = 2.12, d.f. = 4, *p* = .71). With parents’ permission, questionnaires were mailed to the children’s teachers, who returned questionnaires for 94% of children at age 5 years, 93% of those followed to age 7 years, 90% of those followed to age 10 years, and 83% of those followed to age 12 years. The Joint South London and Maudsley and the Institute of Psychiatry Research Ethics Committee approved each phase of the study. Parents gave written informed consent, and twins gave assent between 5 and 12 years of age and then written informed consent at age 18.

### Childhood ADHD Diagnoses

We ascertained childhood ADHD diagnoses on the basis of mother- and teacher-reports of 18 symptoms (9 symptoms of inattention and 9 symptoms of hyperactivity/impulsivity) derived from *DSM-IV* diagnostic criteria.^19^ Meeting diagnostic criteria required 6 or more symptoms of inattention or 6 or more symptoms of hyperactivity/impulsivity reported by mothers or teachers in the past 6 months, and the other informant must have endorsed at least 2 symptoms. We considered participants to have a diagnosis of childhood ADHD if they met criteria at ages 5, 7, 10, or 12. At age 5, 6.6% (n = 138) of the full cohort met criteria for ADHD, 5.4% met criteria at age 7 (n = 109), 3.5% met criteria at age 10 (n = 72), and 3.4% met criteria at age 12 (n = 68). These estimates of ADHD prevalence are within the range found by previous studies.^20^ ADHD symptom levels across childhood were associated with the number of times children met ADHD criteria at the different childhood assessments (see Table S1, available online). Four children taking ADHD medication were added to the childhood ADHD group.

### Age-18 ADHD Diagnosis

We ascertained ADHD diagnosis at age 18 based on private structured interviews with participants regarding 18 symptoms of inattention and hyperactivity/impulsivity according to *DSM-5* criteria.^6^ Participants had to endorse 5 or more inattentive and/or 5 or more hyperactivity/impulsivity symptoms to receive an ADHD diagnosis. We also required symptoms to interfere with the participant’s life at “home, or with family and friends” and at “school or work,” thereby meeting impairment and pervasiveness criteria. The requirement of symptom onset before age 12 was met if parents or teachers reported more than 2 ADHD symptoms at any childhood assessment. A total of 8.1% of participants (n = 166) met criteria for ADHD at age 18. ADHD self-report was corroborated by co-informants at age 18 who reported on 8 ADHD symptoms. A total of 99.3% of participants at age 18 had co-informant ratings, 81.1% from a parent and co-twin, 17.2% from co-twin only, and 1.7% from parent only. Participants with self-reported ADHD at age 18 had more co-informant–rated symptoms than participants without self-reported ADHD (mean = 1.67 versus 0.50, *p* < .001).

### Remitted, Persistent, and Late-Onset ADHD Groups

As reported previously,^6^ we identified 3 groups with ADHD across childhood and young adulthood among participants with information on ADHD in childhood and adulthood (n = 2,040): 9.5% of participants (n = 193) showed remitted ADHD (met diagnostic criteria in childhood but not at age 18), 2.6% (n = 54) showed persistent ADHD (met diagnostic criteria in childhood and at age 18), and 5.5% (n = 112) showed late-onset ADHD (did not meet diagnostic criteria in childhood but did at age 18). The distribution of ethnicity and childhood neighborhood SES among these ADHD groups is provided in Table S2, available online. The rate of ADHD persistence to young adulthood (proportion meeting ADHD criteria at age 18 among participants with childhood ADHD) of 22% is similar to that identified by previous meta-analysis.^2^ Our persistence rate is lower than found in some clinical cohorts or longitudinal studies that select for combined-type ADHD^21^^,^^22^; this is likely due to these studies including more severe ADHD cases. A total of 82.4% of participants (n = 1,681) did not meet criteria for ADHD in childhood or adulthood. Considering the participants with information on genetic risk (total n = 1,838), the different ADHD groups included 9.7% (n = 179) with remitted ADHD, 2.7% (n = 50) with persistent ADHD, and 5.3% (n = 98) with late-onset ADHD; 82.2% (n = 1,511) did not meet criteria for ADHD in childhood or adulthood.

### Genotyping and Imputation

We used Infinium OmniExpress-24 v1.1 BeadChip arrays (Illumina, Inc., San Diego, California) to assay common single nucleotide polymorphism (SNP) variation in the genomes of cohort members. We imputed additional SNPs using IMPUTE2 version 2.3.1 software (https://mathgen.stats.ox.ac.uk/impute/impute_v2.html)^23^ and the 1000 Genomes Phase 3 reference panel.^24^ Imputation was conducted on autosomal SNPs appearing in dbSNP version 140 (http://www.ncbi.nlm.nih.gov/SNP/)^25^ that were “called” in more than 98% of the samples. Invariant SNPs and SNPs with low minor allele frequency (<1%) were excluded. The E-Risk cohort contains monozygotic twins, who are genetically identical; we therefore empirically measured genotypes of one randomly selected twin per pair and assigned these data to their monozygotic co-twin. Monozygotic status was confirmed using genotypic data and SNPs from DNA methylation data for subsets of the sample. We directly measured genotypes of both members of dizygotic twin pairs. Prephasing and imputation were conducted using a 50 million–base pair sliding window. The resulting genotype databases included genotyped SNPs and SNPs imputed with 90% probability of a specific genotype among the European-descent members (90%) of the E-Risk cohort (n = 1,999 participants). Among these, 1,838 participants had information on ADHD in childhood and young adulthood.

### Calculation of PRSs

Polygenic scoring was conducted following the method described by Dudbridge^26^ using PRSice.^27^ Briefly, SNPs reported in the most recent ADHD GWAS^1^ were matched with SNPs in E-Risk, regardless of nominal significance for their association with ADHD. We then performed clumping by retaining the SNP with the smallest *p* value from each linkage disequilibrium block (excluding SNPs with *r*^2^ > .1 in 500-kb windows), then weighted SNPs by effect estimate. To control for possible population stratification, we conducted a principal component analysis of our SNP database using PLINK 1.9^28^ and residualized polygenic scores for the first 10 principal components. The residualized score was normally distributed and standardized to mean zero and SD of 1. PRSs for depression, alcohol dependence, and marijuana use disorder were calculated using the same approach based on the most recent GWASs available for these phenotypes (see Table S3, available online, for additional information).^29^, ^30^, ^31^

### Statistical Analyses

We first tested whether ADHD PRS was associated with ADHD diagnosis at ages 5, 7, 10, and 12 using logistic regressions. We assessed whether ADHD PRS was associated with an increasing number of ADHD diagnoses across childhood using multinomial logistic regression. We then examined the correlation between ADHD PRS and a dimensional measure of total ADHD, hyperactivity/impulsivity, and inattention symptoms as reported by mothers and teachers separately at ages 5, 7, 10, and 12.

Second, we used longitudinal growth models to examine associations between ADHD PRS and hyperactivity/impulsivity and inattention symptom course (separately) across childhood at ages 5, 7, 10, and 12. Because the distribution of ADHD symptoms was highly skewed, we used negative binomial regressions with a multilevel modeling approach in STATA^32^ using the menbreg command. Models included intercepts and linear slopes treated as random effects (ie, allowing individual-level variation), and fixed effects for age and PRS. The main effect of PRS in these models indicates an association with overall level of symptoms across childhood; an interaction between PRS and age indicates PRS is associated with a different slope, or rate of change, of symptoms across childhood.

Third, we examined ADHD course from childhood to young adulthood using multinomial logistic regression to assess whether ADHD PRS was associated with a persistent, remitted, and late-onset course compared with participants who never had ADHD. We tested whether participants with a persistent course had a higher burden of common genetic variants associated with ADHD risk. Additionally, we assessed whether the participants with late-onset ADHD showed similarly elevated PRSs to participants with childhood ADHD to clarify whether late-onset ADHD is genetically similar to childhood ADHD.

Fourth, we repeated these multinomial logistic regressions with PRSs for depression, alcohol dependence, and marijuana use disorder. We investigated whether a higher genetic burden of mental health disorders may be associated with ADHD persistence. We additionally assessed whether late-onset ADHD showed higher genetic risk for these other disorders, possibly suggesting late-onset ADHD may be accounted for by symptoms of other common mental health disorders misidentified as ADHD.

Analyses were corrected for nonindependence of twin observations using the sandwich variance estimator in STATA.^32^

## Results

### Associations Between ADHD Polygenic Risk and Childhood Diagnosis and Symptoms

Children with a higher ADHD PRS were at increased risk of meeting ADHD diagnostic criteria at ages 5, 7, 10, and 12 (Table 1). At age 5, an increase of 1 SD in ADHD PRS was associated with a 45% increased risk of ADHD, and at age 12, a 1 SD increase was associated with a 54% increased risk. ADHD PRS was also associated with number of times a participant met ADHD criteria across childhood: a 1 SD increase in PRS was associated with nearly twice the risk of meeting criteria at 3 or 4 childhood assessments.

**Table 1:** Association of ADHD Polygenic Risk Score With Meeting Childhood ADHD Diagnostic Criteria at Ages 5, 7, 10, and 12 Years and With the Number of Times Meeting Criteria Across Childhood

	N^a^	%	OR	95% CI	*p*
Age					
5	126	6.74	1.45	[1.17, 1.81]	**.001**
7	100	5.51	1.30	[1.04, 1.61]	**.019**
10	69	3.77	1.17	[0.89, 1.53]	.267
12	66	3.67	1.54	[1.21, 1.95]	**< .001**
Number of times meeting criteria across childhood					
0	1,723	87.68	1.00	[reference]	—
1	154	7.84	1.34	[1.09, 1.65]	**.006**
2	60	3.05	1.11	[0.89, 1.39]	.354
3–4	28	1.42	1.92	[1.25, 2.97]	**.003**

Note: Bold *p* values are statistically significant. All standard errors adjusted for twin intracorrelation. ADHD = attention-deficit/hyperactivity disorder; OR = odds ratio.

a N refers to number of participants with polygenic risk score information.

Higher ADHD PRS was also associated with a higher number of ADHD symptoms at ages 5, 7, 10, and 12 (Table 2). Associations between ADHD PRS and symptoms were significant for both mother- and teacher-reports, although of a slightly larger magnitude for mother-reported symptoms. That both mother and teacher ratings showed significant associations suggests both mothers and teachers are sensitive to elevation of symptoms associated with higher genetic risk. Looking separately at the 2 symptom domains, PRS tended to be slightly more strongly associated with hyperactivity/impulsivity than inattention. While correlations were overall significant for each age and symptom reporter, the variance in symptom level explained by ADHD PRS was small.

**Table 2:** Correlations of Attention-Deficit/Hyperactivity Disorder (ADHD) Polygenic Risk Score With Mother- and Teacher-Reported Total ADHD, Hyperactivity/Impulsivity, and Inattention Symptoms in Childhood

	Total symptoms			Hyperactivity/impulsivity			Inattention		
	*r*	*R*^2^^a^ %	*p*	*r*	*R*^2^^a^ %	*p*	*r*	*R*^2^^a^ %	*p*
Mother report									
Age 5	0.10	1.0	**< .001**	0.10	1.1	**< .001**	0.07	0.6	**.004**
Age 7	0.11	1.1	**< .001**	0.12	1.4	**< .001**	0.07	0.5	**.005**
Age 10	0.07	0.4	**.009**	0.07	0.5	**.006**	0.05	0.2	.057
Age 12	0.10	1.0	**< .001**	0.10	1.0	**< .001**	0.08	0.6	**.002**
Teacher report									
Age 5	0.08	0.7	**.003**	0.09	0.7	**.001**	0.06	0.4	**.038**
Age 7	0.06	0.4	**.015**	0.08	0.7	**.001**	0.03	0.1	.249
Age 10	0.06	0.4	**.010**	0.07	0.6	**.003**	0.04	0.2	.118
Age 12	0.09	0.9	**< .001**	0.07	0.5	**.001**	0.09	0.9	**< .001**

Note: Bold *p* values are statistically significant. All standard errors adjusted for twin intracorrelation. *R*^2^ values are derived from linear regression models for ease of interpretability; full results from negative binomial models including McFadden’s pseudo *R*^2^ are presented in Table S5, available online.

a Proportion of variance explained by ADHD polygenic risk scores.

### PRS and Longitudinal Course of Symptoms of Hyperactivity/Impulsivity and Inattention From Childhood to Early Adolescence

Overall symptoms of hyperactivity/impulsivity and inattention declined from early childhood to adolescence. Despite this general decline, children with higher ADHD PRSs showed higher levels of hyperactivity/impulsivity symptoms across childhood (Table 3) compared with children with lower PRSs, of a consistent magnitude from early childhood to adolescence. The same was true for the association with PRS and inattention symptoms across childhood, such that a higher PRS was associated with a consistently higher level of inattention from age 5 to age 12.

**Table 3:** Association of Hyperactivity/Impulsivity and Inattention Mother-Reported Symptoms With Age and Attention-Deficit/Hyperactivity Disorder (ADHD) Polygenic Risk Score Across Childhood

	Main effect model			Interaction with age		
	IRR	95% CI	*p*	IRR	95% CI	*p*
Hyperactivity/impulsivity						
Age	0.83	[0.81, 0.85]	**< .001**	0.83	[0.81, 0.85]	**< .001**
ADHD PRS	1.18	[1.10, 1.26]	**< .001**	1.16	[1.09, 1.24]	**< .001**
ADHD PRS × age				1.01	[0.99, 1.02]	.241
Inattention						
Age	0.91	[0.89, 0.93]	**< .001**	0.91	[0.89, 0.93]	**< .001**
ADHD PRS	1.14	[1.04, 1.24]	**.003**	1.14	[1.04, 1.25]	**.005**
ADHD PRS × age				1.00	[0.98, 1.02]	0.904

Note: Bold *p* values are statistically significant. All standard errors adjusted for twin intracorrelation. IRR = incidence rate ratio; PRS= polygenic risk score.

### Course of ADHD to Young Adulthood and ADHD PRS

**Figure 1:**
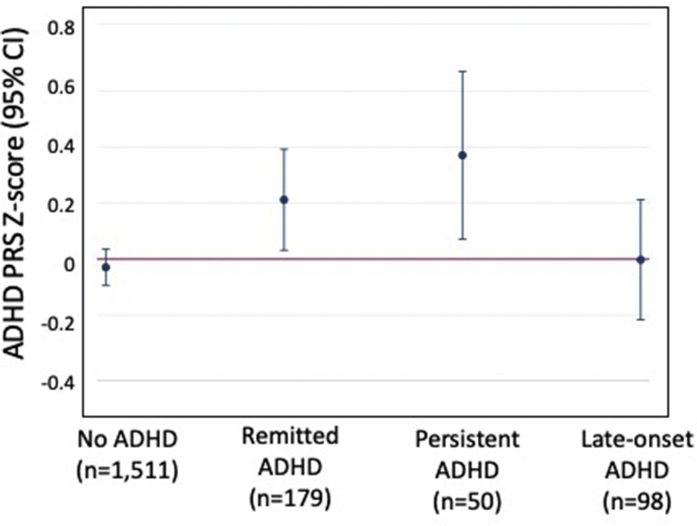
Attention-Deficit/Hyperactivity Disorder (ADHD) Course to Young Adulthood (Remission, Persistence, and Late-Onset) and ADHD Polygenic Risk ***Note:****PRS = Polygenic risk score. Please note color figures are available online.*

We extended our analyses of ADHD course to young adulthood to examine the associations of ADHD PRS with the remission, persistence, and late onset of ADHD by age 18 (Figure 1). The persistent ADHD group had the highest mean ADHD PRS (mean PRS = 0.37), followed by the remitted group (mean PRS = 0.21). However, PRSs in these groups did not significantly differ (*t* = 0.91, d.f. = 1,837, *p* = .37). Participants in the late-onset ADHD group did not show increased ADHD PRS compared with participants who never had ADHD.

### Polygenic Risk for Depression, Alcohol Dependence, and Marijuana Use Disorder and ADHD Course

**Figure 2:**
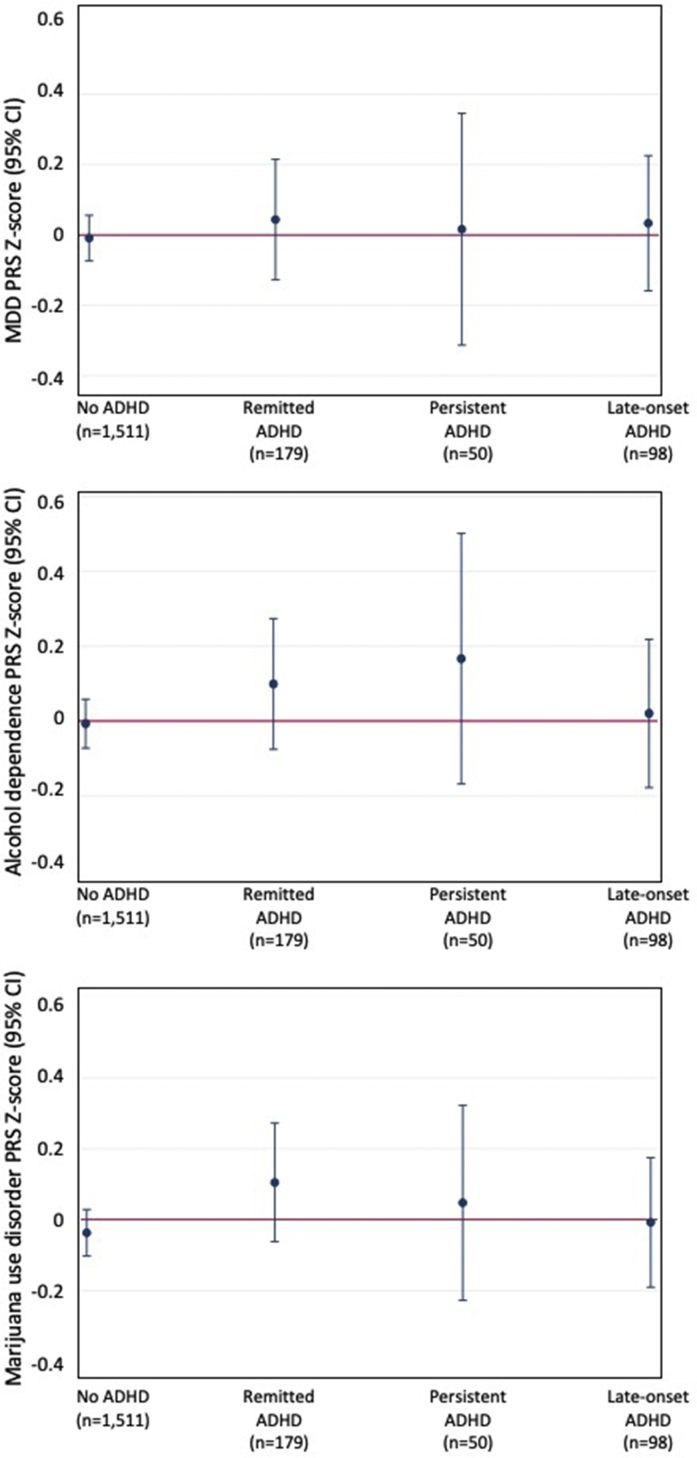
Polygenic Risk for Depression, Alcohol Dependence, and Marijuana Use Disorder and Course of Attention-Deficit/Hyperactivity Disorder (ADHD) (Remission, Persistence, and Late-Onset) at Age 18 ***Note:****Polygenic risk scores (PRSs) for (a) major depressive disorder (MDD), (b) alcohol dependence, and (c) marijuana use disorder. Please note color figures are available online.*

We found that none of the ADHD groups, including participants with late-onset ADHD, showed elevated PRSs for depression (Figure 2a). When examining PRSs for alcohol dependence, participants with persistent ADHD showed somewhat elevated, but nonsignificant, PRSs compared with participants who never had ADHD (*b* = 0.17, 95% CI [−0.17, 0.51]); participants with late-onset ADHD showed no elevation in PRSs for alcohol dependence (Figure 2b). Similarly, none of the ADHD groups showed elevated genetic risk for marijuana use disorder (Figure 2c).

## Discussion

ADHD is increasingly being understood not only as a childhood disorder but also as a dynamic syndrome with differing developmental courses across the life span. We found that higher genetic risk for ADHD was consistently associated with diagnosis and symptom levels at ages spanning early childhood to adolescence. These findings indicate that genetic risk derived from large case-control GWASs can be informative, not only about incidence of ADHD but also about risk for elevated symptoms across childhood. Looking at the course of ADHD diagnosis into young adulthood, participants with persistent ADHD had the highest PRSs; however, PRS did not significantly distinguish participants whose ADHD would persist from participants whose ADHD would remit by age 18. This suggests the need for larger sample sizes as well as a possible role for environmental risk factors that could impact ADHD persistence to adulthood. We did not identify participants in the late-onset ADHD group as having elevated ADHD PRSs compared with controls, indicating genetic risk derived from a largely clinical and pediatric-focused ADHD GWAS is not informative about risk for late-onset ADHD.

### ADHD PRSs Most Elevated Among Participants With Persistent ADHD in Young Adulthood

Participants with persistent ADHD into adulthood had the highest ADHD PRSs among the different developmental patterns. This suggests that genetic factors associated with ADHD case status are also associated with a more persistent course of the disorder over time. ADHD PRSs in participants in the persistent ADHD group may also be elevated owing to the type of cases included in the GWAS used to create the PRS—as some adult cases are included in the GWAS, the PRS may contain variants associated with ADHD persistence.^1^

While most elevated in the persistent ADHD group, the ADHD PRS did not significantly distinguish participants whose ADHD would persist from participants who remitted. This is consistent with findings from a United Kingdom cohort in which ADHD PRSs of participants with high mother-reported ADHD symptoms at age 7 and age 17 were higher than PRSs of controls, but did not significantly differ from the PRSs of participants who had high ADHD symptoms at age 7 only.^33^ While most studies comparing persistent with remitted ADHD groups are limited by small sample sizes, the effect size comparing the persistent and remitted ADHD groups was fairly small (*d* = 0.15). Persistence and remission of ADHD may be influenced by other genetic factors not reflected in the current GWAS that focuses on case-control status. Longitudinal twin studies suggest different genetic influences on level of ADHD symptoms in early childhood and change in symptoms to age 16.^10^ A GWAS focusing on ADHD persistence may be necessary to identify these novel genetic influences on ADHD course. Additionally, environmental factors may play an important role in persistence.

### ADHD PRS Not Associated With Late-Onset ADHD in Young Adulthood

We did not identify a similar profile of genetic risk in the late-onset and childhood-onset ADHD groups. Rather, the ADHD PRS was not elevated among participants with late-onset ADHD. Despite this group meeting full ADHD symptom and impairment criteria at age 18, the PRSs in this group were more similar to non-ADHD controls than participants with remitted or persistent ADHD. Two studies that examined ADHD polygenic risk in adult-onset^4^ and adolescent-onset^33^ ADHD with a PRS derived from an earlier GWAS^34^ also did not identify any significant associations. It is notable that our findings using self-report to identify late-onset ADHD are consistent with findings assessing late-onset ADHD based on parent report.^33^ A recent study examining this question, also based on parent-reported symptoms, found participants with youth-onset ADHD had lower ADHD PRSs than non-ADHD controls.^35^

Our findings add new evidence to support late-onset ADHD having a different underlying genetic composition from childhood-onset ADHD. On one hand, if late-onset ADHD reflects fundamentally the same disorder as childhood-onset ADHD, with the exception that it was undetected in childhood, we would expect these ADHD courses to exhibit a similar genetic risk. On the other hand, if late-onset ADHD does not directly develop from childhood-onset ADHD, it is sensible that a PRS derived from a GWAS largely consisting of cases from childhood clinical populations^1^ may not be elevated among cases in which the disorder was not exhibited in childhood. Thus, our findings offer additional evidence that late-onset ADHD is not simply childhood-onset ADHD that was missed in younger years. Future research could examine whether PRSs derived from GWASs of adult ADHD cases or among cases drawn from population-based cohorts may be more elevated among cases with late-onset ADHD. Additionally, late-onset ADHD may be less genetically driven than childhood-onset ADHD. That environmental factors may play a larger role in adult ADHD is supported by prior twin analyses in this cohort that showed adult ADHD to be less heritable than childhood ADHD.^6^

### ADHD PRS Associated With Consistently Higher Levels of ADHD Symptoms Across Childhood and Into Adolescence

ADHD PRS was associated with more ADHD symptoms at each age, supporting the idea that genetic influence occurs across the dimension of ADHD symptoms in the population. Studies have found that ADHD symptoms tend to decline overall in the population from early to later childhood,^36^ and we found this to be evident in our study with the count of hyperactivity/impulsivity and inattention symptoms declining with age. Despite this overall decline, higher ADHD PRS was consistently associated with elevated ADHD symptoms: participants with higher PRSs showed elevated symptom levels compared with participants with lower PRSs, of a similar magnitude from early childhood to adolescence. However, while there was a significant association between ADHD PRS and symptom level, overall variation in symptom count explained by ADHD PRS was very small, up to 1.4% of the variance in mother-reported hyperactivity/impulsivity symptoms. That a relatively small amount of variance in symptoms is explained by the PRS has been found in other studies; for example, a recent study in a clinical ADHD cohort found the PRS explained 1.8%–2.9% of variance in ADHD symptom level.^11^

Separately examining hyperactivity/impulsivity and inattention symptoms showed that the association with ADHD PRS was slightly stronger for hyperactivity/impulsivity symptoms than for inattention symptoms. These findings are similar to a study of a clinical ADHD population that found a larger difference in levels of hyperactivity symptoms than inattention symptoms between high and low PRS groups.^11^ One explanation for this could be that genetic risk identified by the PRS derived from the current ADHD GWAS is more related to hyperactivity/impulsivity symptoms than inattention symptoms. Children exhibiting externalizing-type problems are more likely to come to clinical attention, leading to a higher likelihood of these children being included in pediatric clinical populations.^37^ Given that many cases in the ADHD GWAS are based on clinical populations, these types of ADHD symptoms may be overrepresented in the GWAS; thus, ADHD PRSs may be more likely to identify externalizing-type ADHD symptoms in independent samples.

### ADHD Course to Young Adulthood Not Associated With Elevated Genetic Risk for Depression, Alcohol Dependence, or Marijuana Use Disorder

We did not find that participants in the persistent ADHD group showed elevated genetic risk for depression, alcohol dependence, or marijuana use disorder, indicating that genetic risk for these disorders commonly comorbid with ADHD did not significantly contribute to ADHD persistence to young adulthood. Similarly, participants with remitted ADHD did not differ from controls or participants with persistent ADHD on these PRSs, suggesting that lower genetic risk for these disorders did not promote ADHD remission. Furthermore, the late-onset ADHD group did not show elevated genetic risk for these other mental health disorders. While this does not rule out that late-onset ADHD may represent other disorders manifesting with symptoms that mimic ADHD, it does suggest that this group does not share the same genetic risk as cases of depression, alcohol dependence, or marijuana use disorder that are included in the GWASs for these disorders.

This study has several strengths including measurement of ADHD diagnosis as well as hyperactivity/impulsivity and inattention symptoms at multiple ages spanning early childhood to young adulthood in a representative population-based cohort. However, our results must be considered in light of some limitations. First, ADHD diagnoses at age 18 were based on self-reports. However, prior work in this cohort found that co-informant reports of ADHD symptoms at age 18 corroborated self-reports, and participants with self-reported late-onset ADHD had significantly more co-informant–rated symptoms than participants without ADHD.^6^ Second, our sample comprised twins, and results may not generalize to singletons. Nevertheless, the prevalence of ADHD at each age in our cohort is within ranges estimated in other samples.^20^ Third, owing to small sample sizes of girls and women with ADHD, we were not powered to examine interactions of sex and PRS in ADHD course. Fourth, rates of ADHD medication use in E-Risk are low (<1%, in line with rates in the United Kingdom^38^), and information on behavioral treatment was not collected in childhood. Therefore, we could not assess whether ADHD treatment was associated with remission. Fifth, the association between ADHD PRS and course could be confounded by indirect genetic effects (eg, higher parental ADHD PRS associated with lower childhood SES). However, adjustment for childhood SES only slightly attenuated results (Table S4, available online). Sixth, we derived PRSs from GWASs performed on samples largely of European ancestry and applied the PRSs to E-Risk participants who were of European ancestry; thus, our results may not generalize to other genetically diverse populations. Additionally, while we statistically adjusted for 10 ancestry principal components to account for population stratification, we cannot rule out the possibility that residual population structure could bias results.

Higher ADHD PRS was associated not only with risk for ADHD diagnosis and elevated symptom levels across childhood but also with a modestly increased risk of ADHD persistence to young adulthood. Yet, ADHD PRS did not differentiate between persistent and remitted ADHD. While higher PRSs among participants with persistent ADHD are suggestive of a higher genetic load in this group, currently ADHD PRS would not be useful in a clinical setting to predict, at an individual level, which child will have persistent ADHD up to young adulthood. However, GWASs of ADHD continue to expand in sample size and diversity of study populations across age, ethnicity, and recruitment from nonclinical settings. This offers the opportunity to further subgroup the ADHD population by different phenotypic presentations and developmental courses, thereby increasing possibilities to identify genetic factors specific to persistence, remission, and late onset. Additionally, further research can investigate how genetic risk may combine with environmental factors to impact the course of ADHD over development. While genes do not change over the life span, the effects of genes on health and behavior are not static and can impact development differentially over time. Quantifying genetic risk through PRS approaches can help us better understand, and someday perhaps predict, the course of mental health disorders over the life span.
