# Supplementary material for: Polygenic Risk and the Course of Attention-Deficit/Hyperactivity Disorder From Childhood to Young Adulthood: Findings From a Nationally Representative Cohort
Source: J Am Acad Child Adolesc Psychiatry. 2021 Sep;60(9):1147–56. doi: 10.1016/j.jaac.2020.12.033 (PMC8417462; doi:10.1016/j.jaac.2020.12.033)
Supplement: Supplemental Material [file mmc1.docx]

**Supplement 1**

**Study cohort full description**

Participants in the cohort were members of the Environmental Risk (E-Risk) Longitudinal Twin Study, a birth cohort of 2,232 British children drawn from a larger register of twins born in England and Wales in 1994-1995.^1^ Details are reported elsewhere.^2^ The E-Risk sample was constructed in 1999-2000, when 1,116 families (93% of those eligible) with same-sex 5-year-old twins participated in home-visit assessments. This sample comprised 56% monozygotic (MZ) and 44% dizygotic (DZ) twin pairs; sex was evenly distributed within zygosity (49% male). Families were recruited to represent the UK population with newborns in the 1990s on the basis of residential location throughout England and Wales and mother’s age. Teenaged mothers with twins were over-selected to replace high-risk families selectively lost to the register through non-response. Older mothers having twins via assisted reproduction were under-selected to avoid an excess of well-educated older mothers. The study sample represented the full range of socioeconomic conditions in the UK, as reflected in families’ distribution on a neighborhood-level socioeconomic index: 25.6% of E-Risk families live in “wealthy achiever” neighborhoods compared to 25.3% nationwide; 5.3% vs. 11.6% live in “urban prosperity” neighborhoods; 29.6% vs. 26.9% live in “comfortably off” neighborhoods; 13.4% vs. 13.9% live in “moderate means” neighborhoods, and 26.1% vs. 20.7% live in “hard-pressed” neighborhoods.^3^ E-Risk underrepresents “urban prosperity” households because they are likely to be childless.

Follow-up home visits took place when study participants were aged 7 (98% participation), 10 (96%), 12 (96%), and 18 years (93% participation). Home visits at ages 5-12 assessed twin participants and their mothers; only twins were assessed at age 18.

Each twin was assessed by a different interviewer. Data are supplemented by searches of official records and questionnaires that are mailed, as developmentally appropriate, to teachers, and co-informants nominated by participants. The Joint South London and Maudsley and the Institute of Psychiatry Research Ethics Committee approved each study phase. Parents gave informed consent and twins gave assent between 5-12 years and then informed consent at age 18.

**Figure S1**

_
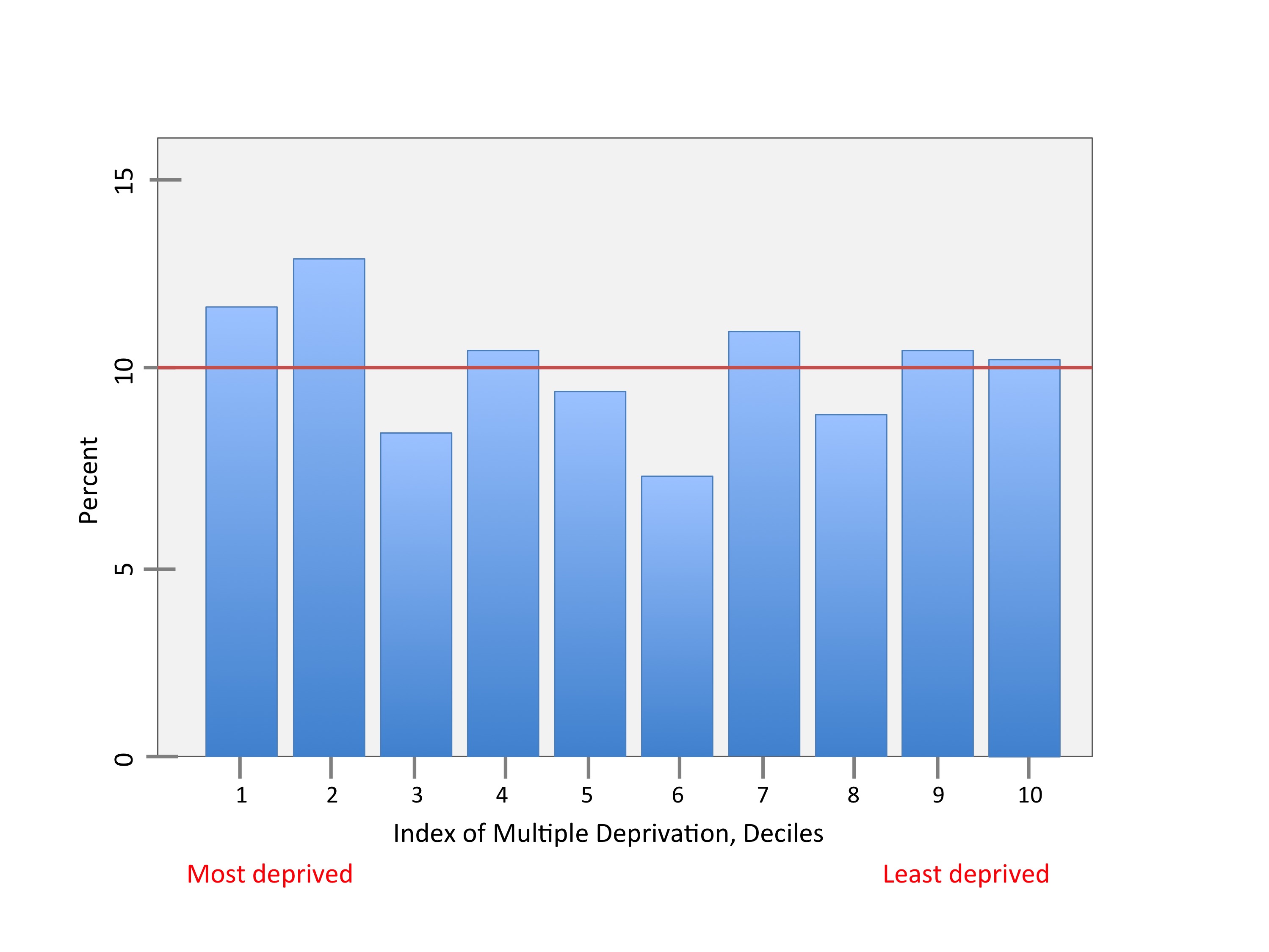
_

**Population representativeness:** The **histogram** shows E-Risk families’ addresses are a near-perfect match to the deciles of the UK’s 2015 Lower-layer Super Output Area (LSOA) Index of Multiple Deprivation (IMD) which averages 1,500 residents; approximately 10% of the cohort fills each of IMD’s 10% bands for the UK. Today, on a neighbourhood-level socioeconomic index called ACORN [A Classification of Residential Neighborhoods, CACI Ltd.], 26% of E-Risk lives in “wealthy achiever” neighbourhoods, compared with 25% nationwide; 5% vs. 12% in “urban prosperity” neighbourhoods; 30% vs. 27% in “comfortably off” neighbourhoods; 13% vs. 14% in “modest means” neighbourhoods; and 26% vs. 21% in “hard-pressed” neighbourhoods. (“Urban prosperity” families are under-represented because they are often childless.)

**E-Risk Genotyped Data**

Mental disorder risk alleles were identified from the studies listed below (Table S3). PGC SNPs were limited to those that passed an imputation quality control threshold akin to that set for the target sample (INFO score >=0.7). Autosomal SNPs that were present in both the target and discovery sample were limited to those in relative linkage equilibrium using the --clump command in PLINK with an R^2^ threshold of .10 and a distance threshold of +/-500kb. These were used to generate polygenic risk scores calculated as the mean number of risk alleles weighted by effect size (log odds ratio). To control for possible population stratification, we conducted a principal components analysis of our genome-wide SNP database using PLINK v1.9 and residualized polygenic scores for the first ten principal components estimated from the genome-wide SNP data. The residualized score was normally distributed and standardized to mean of zero and standard deviation of one.

**Table S1.** Mean ADHD symptoms at ages 5, 7, 10 and 12 among those meeting ADHD criteria at 3 or 4, 2, 1 or 0 times across childhood

|  | Mean mother-reported ADHD symptoms | | | |
| --- | --- | --- | --- | --- |
| # of times meeting ADHD diagnostic criteria in childhood | Age 5 | Age 7 | Age 10 | Age 12 |
| 3-4 | 11.3 | 12.6 | 11.7 | 11.6 |
| 2 | 8.1 | 8.3 | 7.6 | 7.7 |
| 1 | 7.7 | 6.5 | 5.9 | 5.3 |
| 0 | 2.7 | 1.9 | 1.5 | 1.4 |

**Table S2. ADHD group (remitted, persistent, late-onset and never ADHD) by ethnicity and neighborhood-level socioeconomic status**

|  | **Never ADHD** | **Remitted** | **Persistent** | **Late-onset** |
| --- | --- | --- | --- | --- |
| **Ethnicity** | **N (%)** | **N (%)** | **N (%)** | **N (%)** |
| White | 1,513 (90.0) | 179 (92.8) | 52 (96.3) | 100 (89.3) |
| Asian | 72 (4.3) | 6 (3.1) | 1 (1.9) | 5 (4.5) |
| Black | 31 (1.8) | 4 (2.1) | 1 (1.9) | 4 (3.6) |
| Mixed race | 6 (0.4) | 2 (1.0) | 0 (0.0) | 0 (0.0) |
| X^2^=11.3, p=0.51 | | | | |
| **Neighborhood SES at age 5^a^** | **N (%)** | **N (%)** | **N (%)** | **N (%)** |
| Wealthy achievers | 390 (23.5) | 38 (20.1) | 12 (24.0) | 16 (15.1) |
| Urban prosperity | 93 (5.6) | 9 (4.8) | 0 (0.0) | 7 (6.6) |
| Comfortably off | 493 (29.7) | 43 (22.8) | 9 (18.0) | 33 (31.1) |
| Moderate means | 242 (14.6) | 32 (16.9) | 9 (18.0) | 14 (13.2) |
| Hard pressed | 440 (26.5) | 67 (35.5) | 20 (40.0) | 36 (34.0) |
| X^2^=22.2, p=0.04 | | | | |

1. Neighbourhood-level socioeconomic is categorized with the ACORN Index [A Classification of Residential Neighborhoods, CACI Ltd.].

**Table S3.** PRS references GWAS and number of SNPs matched to E-Risk genotype data

| **PRS** | **Source (reference)**  **N cases/controls**  **nSNPS in GWAS summary statistics** | **nSNPS matched in genotype data (out of 5,158,026 available)** |
| --- | --- | --- |
| **ADHD** | Demontis et al 2019 *Nat Genetics*  20,183/35,191  8,047,421 SNPs | 4,481,675 |
| **Depression** | Wray et al. 2018 *Nat Genetics*  135,458/344,901  13,554,550 SNPs | 4,397,836 |
| **Alcohol dependence** | Walters et al 2018 *Nat Neurosci*  14,904/37,944  9,225,633 SNPs | 4,435,116 |
| **Marijuana use disorder** | Demontis et al 2019 *Nat Neurosci*  2,387/48,985  8,971,679 SNPs | 4,449,260 |

**Table S4.** Multinomial logistic regression of ADHD course (remission, persistence and late-onset) compared with those who never had ADHD, unadjusted and adjusting for childhood socioeconomic status

|  | **Remitted vs never ADHD** | | **Persistent vs never ADHD** | | **Late-onset vs never ADHD** | |
| --- | --- | --- | --- | --- | --- | --- |
|  | RR (95% CI) | | RR (95% CI) | | RR (95% CI) | |
|  | Unadjusted | Adjusted | Unadjusted | Adjusted | Unadjusted | Adjusted |
| **ADHD PRS** | 1.27* (1.1, 1.5) | 1.24* (1.0, 1.5) | 1.49* (1.1, 2.0) | 1.46* (1.1, 2.0) | 1.03 (0.8, 1.3) | 1.01 (0.8, 1.2) |
| **Child SES** |  |  |  |  |  |  |
| **Low** |  | 2.55*** (1.6, 4.0) |  | 1.78 (0.8, 3.9) |  | 1.98* (1.1, 3.4) |
| **Medium** |  | 1.36 (0.8, 2.2) |  | 0.92 (0.4, 2.1) |  | 1.32 (0.7, 2.4) |
| **High** |  | 1.00 (ref) |  | 1.00 (ref) |  | 1.00 (ref) |

**Table S5.** Negative binomial models of the association between ADHD PRS and total, hyperactivity/impulsivity and inattention symptoms in childhood as reported by mothers and teachers

|  | *Total symptoms* | | | *Hyperactivity/ impulsivity* | | | *Inattention* | | |
| --- | --- | --- | --- | --- | --- | --- | --- | --- | --- |
| *Mother-report* | *b* | *Pseudo R^2^* | *p value* | *b* | *Pseudo R^2^* | *p value* | *b* | *PseudoR^2^* | *p value* |
| Age 5 | 0.11 | 0.2% | **<0.001** | 0.11 | 0.2% | **<0.001** | 0.12 | 0.2% | **0.004** |
| 7 | 0.14 | 0.2% | **<0.001** | 0.15 | 0.3% | **<0.001** | 0.13 | 0.2% | **0.005** |
| 10 | 0.11 | 0.1% | **0.009** | 0.12 | 0.1% | **0.006** | 0.09 | 0.1% | 0.057 |
| 12 | 0.16 | 0.2% | **<0.001** | 0.17 | 0.3% | **<0.001** | 0.14 | 0.2% | **0.002** |
| *Teacher-report* | *b* | *Pseudo R^2^* | *p value* | *b* | *Pseudo R^2^* | *p value* | *b* | *PseudoR^2^* | *p value* |
| Age 5 | 0.21 | 0.2% | **0.003** | 0.26 | 0.4% | **0.001** | 0.17 | 0.2% | **0.038** |
| 7 | 0.18 | 0.1% | **0.015** | 0.30 | 0.4% | **0.001** | 0.09 | 0.04% | 0.249 |
| 10 | 0.21 | 0.2% | **0.010** | 0.29 | 0.4% | **0.003** | 0.14 | 0.1% | 0.118 |
| 12 | 0.36 | 0.5% | **<0.001** | 0.32 | 0.4% | **0.001** | 0.39 | 0.6% | **<0.001** |

b= regression coefficient; pseudo R^2^=McFadden's pseudo R-squared

References

1. Trouton A, Spinath F, Plomin R. Twins Early Development Study (TEDS): a multivariate, longitudinal genetic investigation of language, cognition and behavior problems in childhood. Twin Res*.* 2002;5:444-448.

2. Moffitt TE, E-Risk Study Team. Teen-aged mothers in contemporary Britain. J Child Adolesc Psychiatry*.* 2002;43:727-742.

3. Odgers C, Caspi A, Russell M, Sampson R, Arseneault L, Moffit TE. Supportive parenting mediates neighborhood socioeconomic disparities in children's antisocial behavior from ages 5 to 12. Developmental Psychopathology*.* 2012;24:705-721.
